# The 3,550 g Challenge: An Atypical Presentation of a 3.5 kg Seminoma Manifesting as Dry Skin

**DOI:** 10.7759/cureus.87174

**Published:** 2025-07-02

**Authors:** Nicolas Omorphos, Mohamed Shamil Mohsin, Spencer Mok, Mark Kitchen, Kuo J Ho

**Affiliations:** 1 Urology, Heartlands Hospital, Birmingham, GBR; 2 Urology, Royal Shrewsbury Hospital, Shrewsbury, GBR; 3 Urology, University Hospitals Birmingham NHS Foundation Trust, Birmingham, GBR

**Keywords:** adjuvant chemotherapy, auc, radical orchidectomy, serum tumor markers, testicular seminoma

## Abstract

A 43-year-old male presented to the emergency department with a seven-year history of progressive left-sided scrotal swelling. On examination, a large mass was palpable in the left hemiscrotum, accompanied by eczema-like skin changes and a decubitus ulcer. Tumor markers were significantly elevated, particularly lactate dehydrogenase, and an urgent ultrasound confirmed the presence of a testicular tumor. CT revealed a scrotal mass originating from the left testis, along with bilateral inguinal lymphadenopathy. The patient underwent a left inguinal orchidectomy, and histopathological analysis confirmed a 3,550 g classical seminoma. He was subsequently referred to oncology for adjuvant chemotherapy.

## Introduction

Testicular cancers are relatively rare, comprising approximately 1% of all adult malignancies and about 5% of urological tumors. Their prevalence in Western societies is estimated at three to 10 cases per 100,000 males per year. In recent decades, particularly in industrialized countries, the incidence of testicular cancer has been on the rise. At the time of diagnosis, the majority of cases are germ cell tumors (GCTs), which account for 90-95% of all testicular cancers [1]. Most cases present as unilateral, firm testicular masses, typically identified through self-examination or during radiological investigations for scrotal swelling or pain.

According to the 2022 WHO classification, testicular GCTs are categorized into three main groups [2]: (1) GCTs derived from germ cell neoplasia in situ (GCNIS), including GCNIS, seminomas, non-seminomatous GCTs (such as postpubertal teratomas, embryonal carcinomas, yolk sac tumors, and choriocarcinomas), and mixed GCTs; (2) GCTs unrelated to GCNIS, which include spermatocytic tumors, prepubertal teratomas and yolk sac tumors, and neuroendocrine tumors; and (3) sex cord-stromal tumors, encompassing Leydig cell tumors, Sertoli cell tumors, and mixed stromal tumors.

Non-seminomatous testicular cancers and mixed GCTs most commonly occur in the third decade of life, whereas seminomas peak in the fourth decade. Seminomas account for approximately 50% of all GCTs and are generally characterized by slow growth [2].

## Case presentation

A 43-year-old male presented to the emergency department with a seven-year history of gradually increasing left-sided scrotal swelling, associated with overlying skin dryness, rash, and a decubitus ulcer, the latter being the primary reason for his initial consultation with his general practitioner (GP). The patient did not appear to recognize the significance of the scrotal swelling. Prior to his emergency presentation, his GP had arranged an ultrasound scan and initiated a two-week wait referral via the suspected cancer pathway. His past medical history included asthma, obesity, and well-controlled hypertension. He had no relevant surgical history.

On initial assessment, the patient was noted to be overweight, with a grossly enlarged scrotum, prominent tortuous superficial veins, and dry skin. A decubitus ulcer was also present. Due to the size of the left testicular mass, neither testis was palpable separately. Baseline blood tests, including serum testicular tumor markers, were performed, and imaging was arranged, including a scrotal ultrasound and staging CT of the thorax, abdomen, and pelvis. Laboratory results revealed a significantly elevated lactate dehydrogenase (LDH) level of 2,425 U/L (reference range: 125-220 U/L) and a mildly elevated beta-human chorionic gonadotropin (β-hCG) level of 26 IU/L (reference range: <2.4 IU/L).

**Figure 1:**
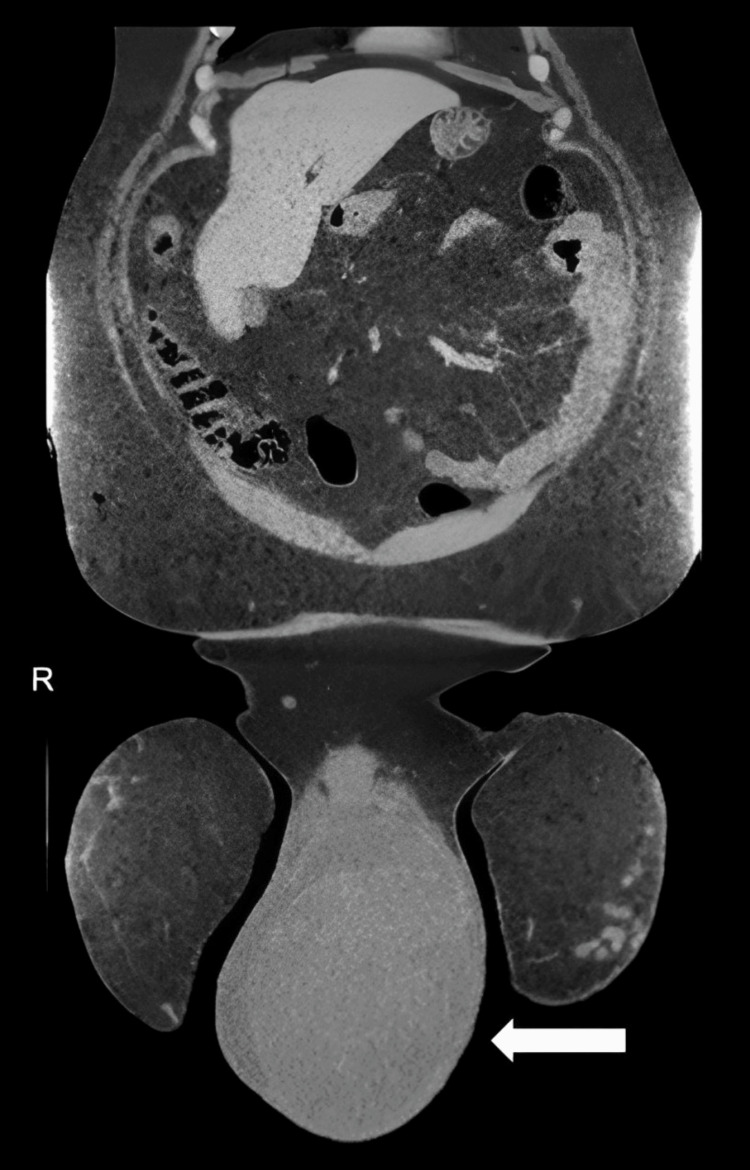
Coronal CT image showing the testicular mass

A consultant radiologist performed the scrotal ultrasound, which demonstrated a normal-sized right testicle with good vascularity and a grossly enlarged left testicle measuring approximately 16 cm in diameter. The left testis showed abnormal echotexture consistent with a large testicular tumor, along with a thin rim of surrounding fluid. Staging CT confirmed a 16 × 16 cm scrotal mass arising from the left testis, accompanied by a small adjacent hydrocele. Mildly enlarged inguinal lymph nodes were noted bilaterally - more prominent on the left - and a 1.5 cm left para-aortic lymph node was identified, although its clinical significance remained uncertain (Figure 1).

**Figure 2:**
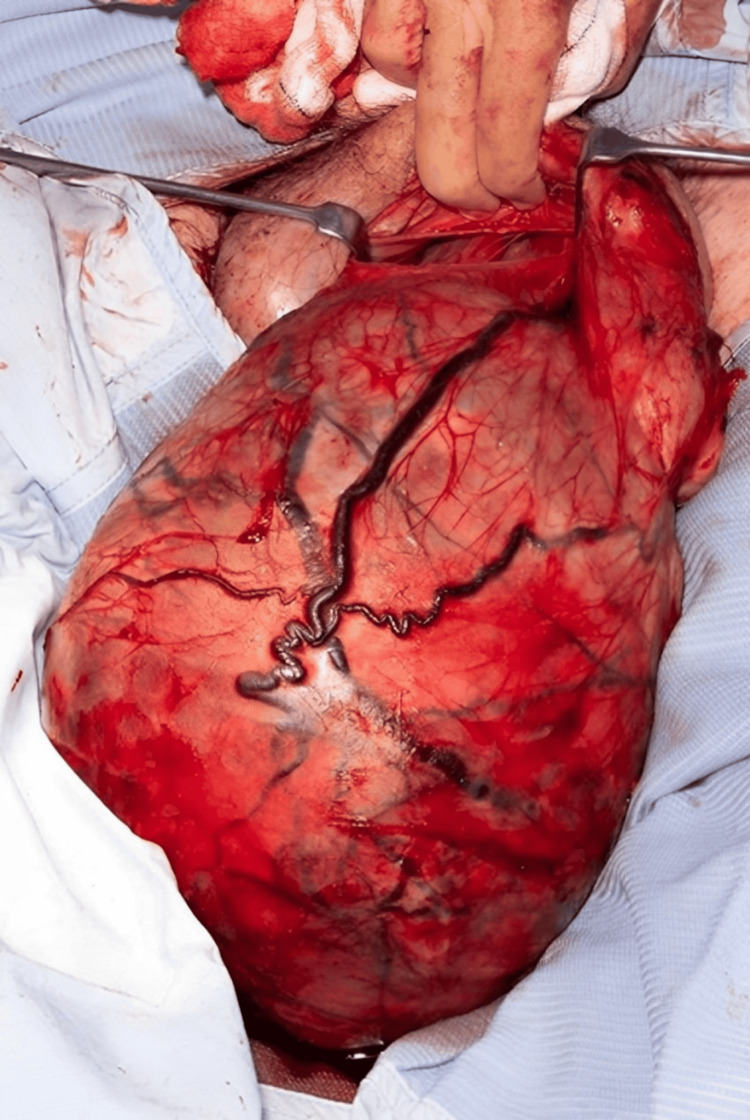
Orchidectomy performed via a scrotal approach, with the testicle visible within the tunica vaginalis

**Figure 3:**
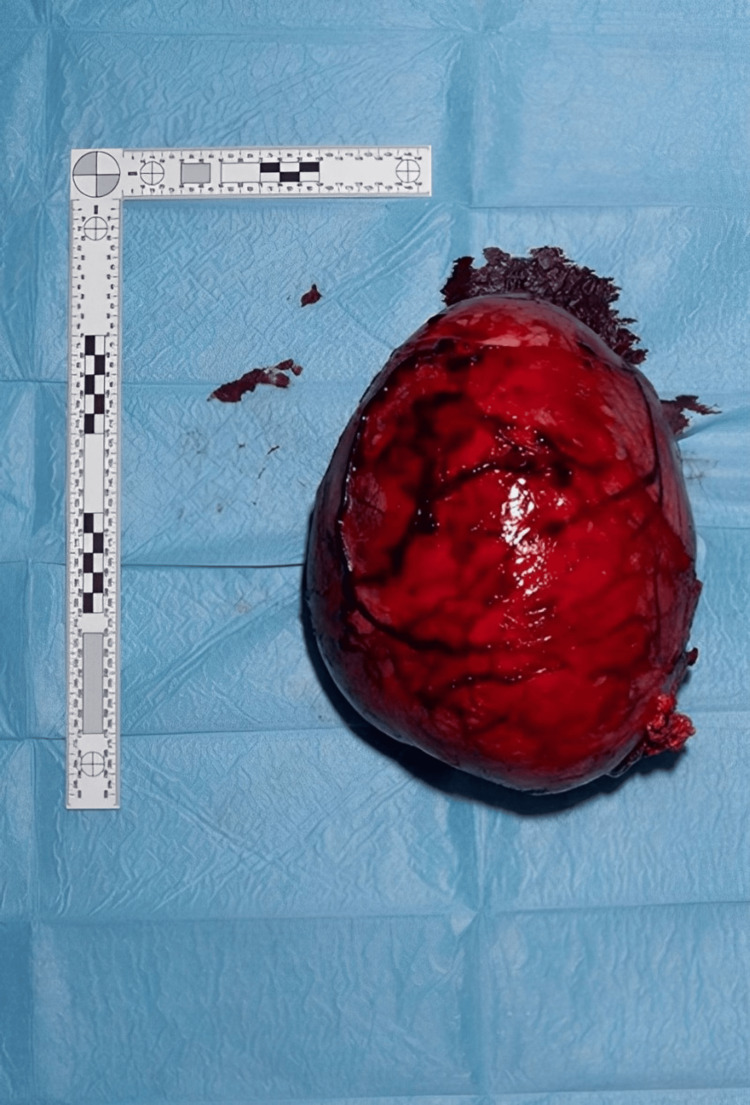
Orchidectomy specimen

Due to the size of the mass, a radical orchidectomy was performed via a scrotal rather than the standard inguinal approach (Figure 2, Figure 3). The patient recovered well post-operatively and was subsequently discharged home. Histological analysis confirmed a 3,550 g classical seminoma measuring 350 × 300 × 200 mm. The tumor occupied the entire testis and showed areas of hemorrhage, necrosis, and cyst formation, with extensive regions of infarction. The tumor was staged as pT2. Immunohistochemical staining demonstrated weak but positive expression of CD117, OCT3/4, and PLAP, while CAM5.2, CD30, and alpha-fetoprotein (AFP) were negative.

The urology cancer multidisciplinary team recommended immediate adjuvant chemotherapy, and the patient was initiated on adjuvant carboplatin, dose-adjusted according to renal function (AUC 7 protocol). As part of the follow-up, restaging CT scans of the thorax, abdomen, and pelvis were performed at one and eight months post-operatively. The one-month follow-up scan demonstrated a reduction in the size of the left para-aortic lymph node (7 mm from 15 mm), sub-centimeter external iliac lymph nodes, and stable but enlarged inguinal nodes (1.8 cm on the left and 0.9 cm on the right). The eight-month scan showed a barely discernible left para-aortic node, persistently enlarged inguinal nodes, and no evidence of new metastatic lesions.

Tumor markers were reassessed at three weeks and eight months following the orchidectomy (Table 1).

**Table 1:** Comparison of tumor marker levels in the preoperative period and at three-week and eight-month postoperative follow-ups AFP, alpha-fetoprotein; β-hCG, beta-human chorionic gonadotropin; FSH, follicle-stimulating hormone; LDH, lactate dehydrogenase; LH, luteinizing hormone; SHBG, sex hormone-binding globulin

Marker	Reference range	Preoperative	Three weeks postoperative	Eight months postoperative
AFP	0-7 kU/L	3.6	3.5	3.4
LDH	125-220 U/L	2,425	258	245
β-hCG	<2.4 IU/L	26	<1	<2
Testosterone	7-27 nmol/L	13.7	-	-
SHBG	14-71 nmol/L	49	-	-
LH	0.6-12.1 IU/L	9.5	-	11.4
FSH	1-12.0 IU/L	11	-	11.7

## Discussion

Seminomas are a type of germ cell testicular tumor and can be classified into three histological variants [3,4]: (1) classical/pure seminoma (approximately 85% of cases) - the most common subtype; (2) anaplastic seminoma (seen in about 10% of patients); and (3) spermatocytic seminoma (accounts for around 5% and typically affects older men over 60 years).

Pure seminomas are the most frequently occurring variant, primarily affecting men in their fourth decade of life. The tumor is believed to develop from totipotent germ cells that undergo abnormal differentiation rather than maturing into spermatocytes through the typical developmental pathway. Clinically, patients usually present with a painless, slow-growing testicular mass. However, acute scrotal pain occurs in approximately 10% of cases, likely due to intratesticular hemorrhage or infarction. The mass is often hard, distinct from the scrotal skin, and may be accompanied by a hydrocele.

Serum tumor markers commonly assessed include AFP, LDH, and β-hCG. Notably, elevated AFP essentially rules out pure seminomatous disease. LDH, while less specific, correlates with overall tumor burden. β-hCG is elevated in 5-10% of seminoma cases and may indicate metastatic spread, although it does not appear to influence overall survival [5].

Management of seminomas typically begins with radical inguinal orchidectomy, which serves both diagnostic and therapeutic purposes. In Stage I disease, orchidectomy alone results in a cure in approximately 80% of cases. To reduce recurrence risk, adjuvant therapy may include single-agent carboplatin or radiotherapy. For Stage II disease, treatment is more varied and may involve orchidectomy followed by radiotherapy or chemotherapy with bleomycin, etoposide, and cisplatin (BEP) or etoposide and cisplatin (EP) regimens, depending on local guidelines and individual prognostic factors [1].

Metastatic spread typically follows a predictable stepwise lymphatic pattern. For right-sided tumors, initial spread is to the inter-aortocaval nodes, followed by paracaval, preaortic, and paraaortic lymph nodes. Left-sided tumors usually spread first to the preaortic and paraaortic nodes, then to the inter-aortocaval region. In advanced disease, retrograde metastasis to the common iliac, external iliac, and inguinal lymph nodes may occur. Contralateral spread is more common with right-sided tumors, and bilateral nodal involvement is found in approximately 15-20% of patients.

European guidelines recommend staging CT scans of the thorax, abdomen, and pelvis to evaluate for metastases, particularly in the lungs and retroperitoneum. A PET-CT scan is advised in cases with residual mass; if positive, surgical resection should be considered.

Follow-up protocols as per the European Guidelines include regular clinical examinations and tumor marker assessments (AFP, β-hCG, and LDH) at defined intervals - every three months during year 1, every four months in year 2, every six months until year 5, and annually thereafter. Imaging with CT or MRI, plus chest X-ray, is recommended at three months and at years 1, 2, and 5.

## Conclusions

This report presents what is believed to be the largest seminoma recorded in the current literature. Although delayed presentation may have been influenced by the COVID-19 pandemic, the case underscores the importance of public education, awareness, and self-examination campaigns to promote early detection of testicular tumors. It also highlights the efficacy of modern treatment protocols for seminoma. Despite the exceptionally large tumor burden at diagnosis, the patient showed no signs of disease progression following radical orchidectomy and adjuvant chemotherapy.
